# Machine Learning-Based Classification of the Health State of Mice Colon in Cancer Study from Confocal Laser Endomicroscopy

**DOI:** 10.1038/s41598-019-56583-9

**Published:** 2019-12-27

**Authors:** Pejman Rasti, Christian Wolf, Hugo Dorez, Raphael Sablong, Driffa Moussata, Salma Samiei, David Rousseau

**Affiliations:** 1 Laboratoire Angevin de Recherche en Ingénierie des Systèmes (LARIS), UMR INRA IRHS, Université d’Angers, Angers, 49000 France; 2 INSA-Lyon, INRIA, LIRIS, CITI, CNRS, Villeurbanne, France; 3 Univ Lyon, INSA-Lyon, Université Claude Bernard Lyon 1, UJM-Saint Etienne, CNRS, Inserm, CREATIS UMR 5220, U1206, Lyon, 69621 France

**Keywords:** Cancer imaging, Computer science, Software

## Abstract

In this article, we address the problem of the classification of the health state of the colon’s wall of mice, possibly injured by cancer with machine learning approaches. This problem is essential for translational research on cancer and is a priori challenging since the amount of data is usually limited in all preclinical studies for practical and ethical reasons. Three states considered including cancer, health, and inflammatory on tissues. Fully automated machine learning-based methods are proposed, including deep learning, transfer learning, and shallow learning with SVM. These methods addressed different training strategies corresponding to clinical questions such as the automatic clinical state prediction on unseen data using a pre-trained model, or in an alternative setting, real-time estimation of the clinical state of individual tissue samples during the examination. Experimental results show the best performance of 99.93% correct recognition rate obtained for the second strategy as well as the performance of 98.49% which were achieved for the more difficult first case.

## Introduction

Classically the characterization of colon’s pathology is realized from histology^1^ but is now also investigated with *in vivo* imaging techniques which enable the oncological^2^ early detection of abnormal physiological processes such as inflammation of dysplastic lesions. This includes chromoendoscopy^3^, confocal laser endomicroscopy^4,5^ or multiphoton microscopy^6^. These modern video-microscopies introduced in preclinical studies on mice with the promises of translational research^7^.

These imaging techniques are producing videos which for the inspection of one colon of one mouse corresponds to thousands of frames to be further multiplied by the number of mice inspected. Each frame of these videos can be different in the structure and texture as it is recorded over a colon’s wall with movement of the probe, spurious presence of unexpected items between probes and colon, variation of contrast agent concentration. To draw benefit from such imaging protocols, the bottleneck is thus the automation of the image analysis. In this article, we consider one of these protocols and propose a fully automated solution for the classification of colon wall images into healthy, inflammation and dysplastic tissues.

We work with the confocal endomicroscopy imaging protocol of^5^ for the classification of the health state of the colon’s wall of mice. Since its introduction, this protocol has seen widespread usage in multiple research groups^8–10^. So far, image analysis for the classification of colon’s wall health state with this protocol has been relatively limited. The existing literature is based on handcrafted features^5,8–10^.

In this article, we go beyond the sole characterization (feature handcrafting) and, for the first time on Mice colon in cancer study from confocal laser endomicroscopy, in the growing trend of machine learning applied to medical image analysis^11–13^, propose a fully automated classification method based on supervised learning that we validate on thousands of images. This work is a priori challenging since the amount of data in preclinical studies, such as in our case, is rather limited compared to the usual amount of data available in medical applications of machine learning. Also, another a priori open question addressed in the preclinical study is the question of translational research, i.e. the reusability of the knowledge gained for animals on human or human on animals. We address this question here, for the first time to our knowledge, in the perspective of machine learning. As the last innovation in our methodology to address a specific unsolved preclinical problem, we discuss different scientific use cases and corresponding strategies for training concerning some properties of confocal laser endomicroscopy. Images are acquired at the video frame rate while the expert holding the endoscopic probes moves it slowly to inspect the tissue when located close to the tissue of interest. Consequently, though the imaging system is producing vast amounts of images, a large number of images are very similar. We consider the possibility of taking benefit from this self-similarity in order to significantly reduce the size of the data set requested during the training stage. This training approach is vital for the expert in charge of the annotation of the training data sets since it is a highly time-consuming task. In a second configuration, we also discuss the performance obtained with different machine learning approaches when we learn on images corresponding to a given set of mice while applying the classification on a distinct cohort of mice. This cross-subject training is relevant for clinical purposes because it quantifies to which extend the disease observed is generic or patient-specific. The performances of these two training strategies compared to the best performance obtained with a brute force random sampling on a whole cohort for the training of the classification algorithm.

In the literature, several studies have focused on the classification of colon’s health state from endomicroscopy. Up to our knowledge, this body of work based on the classical methodology of handcrafted feature design (taking into account domain knowledge), followed by supervised machine learning.

A method based on global descriptors proposed in^5^, whose introduced fractal box-counting metrics and illustrated them on two images. Vessel detection was proposed in^8^ after a Hessian-based filter in addition to length area and diameter measurements of vascular crypts of the colon’s wall. Blood vessels of the colon’s wall characterized in^9^ from Fourier analysis. Also, vascular networks of colon’s wall were characterized in terms of graphs in^10^ after skeletonization on few hundreds of images.

**Figure 1:**
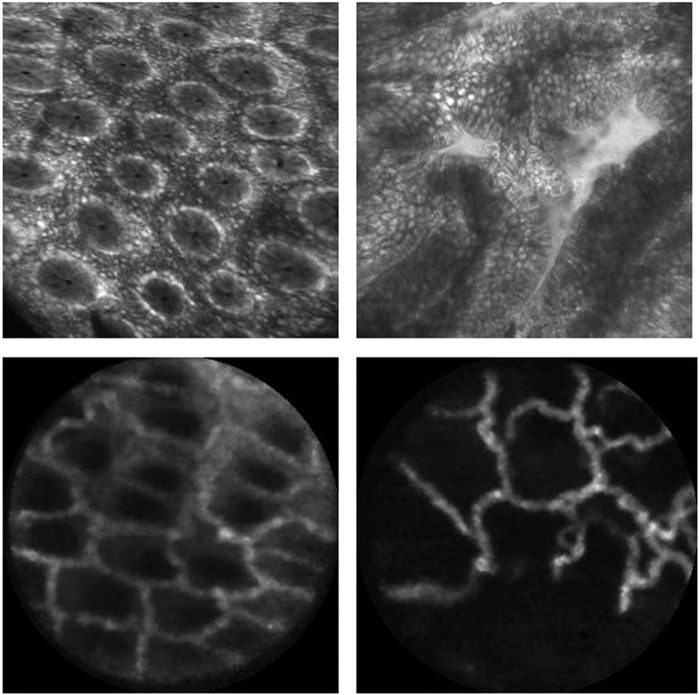
Top: Human samples of colon’s wall images: healthy (left) and unhealthy (right) tissues observed from fluorescent confocal endomicroscopy. Bottom: Mouse samples of colon’s wall images: healthy (left) and unhealthy (right) tissues observed from fluorescent confocal endomicroscopy.

Closest to our work is the method by Ştefănescu *et al*., which is based on machine learning with neural networks of images of human tissues^14^ acquired with confocal laser endomicroscopy. However, the images are clearly different; in contrast, the field of view and resolution, as can be seen in Fig. 1. These differences motivate our proposition of designing a specific method for mice trained on mouse images. In contrast to^14^, we (i) propose a method based on representation learning^15^ as opposed to handcrafted features, and (ii) specifically discuss different experimental protocols and develop different training strategies adapted to these protocols.

## Results

In this section, we give experimental results using the experimental protocol and training strategies described in the method section as well as the different feature extraction and feature learning techniques.

### Cross-subject training

For this protocol, the most challenging one of all considered cases, where generalization to unseen subjects (mice) is required, randomly chosen images of mice for three datasets of training, validation, and testing as shown in Table 1. While the training set is used to adjust the parameters of the model, the validation set is used to minimize overfitting and tune the parameters. The test set of unseen data is used to confirm the predictive power and that the model generalises. The final classification of trials is computed as the average performance of each fold. The number of healthy and unhealthy mice are not equal. We simulated cross-validation for this approach by changing mice between training, validation, and testing for each new experiment.

**Table 1:** Number of mice in each dataset.

	Healthy mice	Mice with cancer	Mice with inflammation
Training	5	7	7
Validation	1	2	2
Testing	3	4	7

Table 2 gives results with the different feature representations and classifiers described in the method section. In addition, Table 3 shows classification accuracy of a transfer learning method with different freezing layers discussed in section. Our proposed architecture trained from scratch shows the best recognition rate compared to handcrafted features, and state of the art high-capacity architectures with pre-training. The experiments indicate that high-capacity networks overfit on this amount of target data even when they are pre-trained on large datasets of natural images. We conjecture that the shift in data distributions is too large in the case of this application. The last layer of the network, still trained from scratch even in the case of transfer learning, overfits on the small target data set. To sum up the essence of the contribution, we train a high-capacity model on a large scale data set, followed by fine-tuning of a low capacity SVM model on the small volume target data set.

**Table 2:** Left: Results of cross-subject training with full data, where all images of 6 healthy mice, 9 mice with cancer, and 9 mice with inflammation used for training the system. Right: Confusion matrix of cross-subject performance where our proposed CNN architecture is used.

Left			Right			
Classifiers	Transfer learning	Accuracy		True Cancer	True Inflammation	True Healthy
Proposed CNN architecture	—	**98.49**% ± 0.6	Predicted Cancer	13107	0	0
DenseNet	X	94.54% ± 2.9	Predicted Inflammation	0	5012	46
VGG16 + linear SVM	X	90.60% ± 0.4	Predicted Healthy	0	75	2011
VGG16	X	89.62% ± 3.3				
ResNet50	X	75.93% ± 4.1				
VGG16	—	74.82% ± 3.2				
LBP features + linear SVM	—	83.01% ± 0.4				
Proposed method at^14^	—	77.41% ± 1.3

**Table 3:** Results of cross-subject training with different numbers of frozen layers when transferring the VGG16 network from ImageNet to the target dataset.

No. Freezing Conv. layers	1	2	3	4	5	6	7	8	9	10	11	12	13
Accuracy	40.8% ± 17.4	65.6 ± 29.9%	89.6 ± 3.3%	89.2% ± 3.9	42.8% ± 21.9	43.4% ± 23.25	70% ± 24.1	52.8% ± 22.2	75.4% ± 23.9	82.2% ± 9.4	65.8% ± 29.9	41.2% ± 18.3	33% ± 0

**Figure 2:**
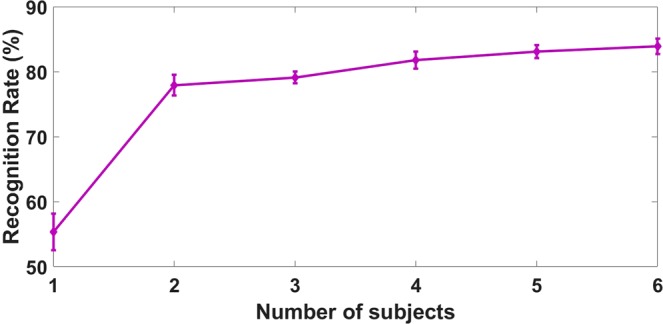
Dependency on the number of training subjects for cross-subject training (LBP features + SVM classifier).

Also, we studied the dependency of the classification results on the number of subjects in the training data, as illustrated in the Fig. 2. For this study, we chose the LBP based representation and the SVM classifier since it can work better when a small size of the database is available for training. As expected, the system performance increases significantly when additional mice are added to the training set, as each mouse potentially has its specific pattern for health, inflammation, and cancer tissues.

**Figure 3:**
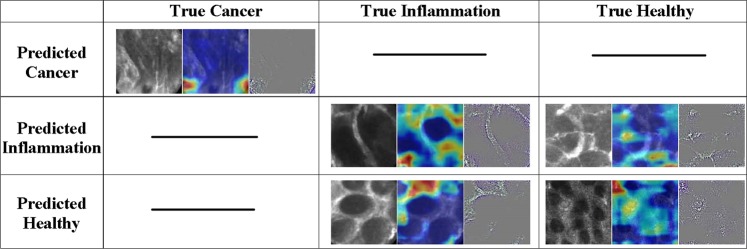
Example of correctly and miss classified images of the proposed CNN architecture for the cross-subject training strategy. Each cell consists from left to right of a grayscale image, a coarse localization map of the important regions in the image for the network^40^, and a high-resolution class-discriminative visualization^40^. Cells with dashed lines mean that there is no miss classified images for that class.

Figure 3 shows some cases of correctly and wrongly classified images with their coarse localization maps. As can be seen, these images are indeed difficult to assess as the miss classified images have a similar pattern with another class.

### Cross-sample training with all samples

Let us recall that in another use case of cross-sample training, subjects (mice) are mixed between training and test sets. In our setup, the 7 fold cross-validation approach used where almost 75% of images are dedicated for training and 25% of images for testing purposes, which corresponds to the proportions chosen for a similar problem in^14^, albeit for human colon’s walls. When needed, the validation set was chosen from the training set. Table 4 gives the prediction performance of the different classifiers on this data. We report means and standard deviations of ten runs.

**Table 4:** Left: Results of cross-sample training with full data. Right: Confusion Matrix of cross-sample performance where our proposed CNN architecture is used.

Left			Right			
Classifiers	Transfer learning	Accuracy		True Cancer	True Inflammation	True Healthy
Proposed CNN architecture	—	**99.93%** ± 0.13	Predicted Cancer	13994	0	0
LBP features + linear SVM	—	97.7% ± 0.39	Predicted Inflammation	0	4032	0
VGG16 + linear SVM	X	85.9% ± 0.4	Predicted Healthy	0	5	1849
VGG16	X	82.12% ± 4.1				
ResNet50	X	79.94% ± 4.6				
DenseNet	X	79.51% ± 3.8				
VGG16	—	78.49% ± 1.27				

In this more natural case, where correlations between subsequent frames in the input video can be exploited, our CNN architecture still outperforms other models and feature learning methods with a close to perfect performance of 99.33%. Even transfer learning of deep networks cannot compete in this section, where generalization to unseen subjects is not an issue. We conjecture that the reason is that pre-training on the large-scale data set learns a representation tailored for high generalization, which requires encoding invariances to large deformation groups into the prediction model. These invariances help to recognize natural classes, like animals and objects from daily life, even though their viewpoints and shapes might be profoundly different. It is clearly not the objective for our cross-sample use case, where generalization is less an issue than encoding extremely fine-grained similarities between samples which are very close in feature space.

Overall deep learning methods with a pre-training, the best results were obtained by the VGG16 model pre-trained on ILSVRC and fine-tuned on our target data set, where after fine-tuning a linear SVM classifier was trained on the last feature layer of the deep network. Interestingly, this performance is comparable to what was obtained in^14^ for a similar colon’s wall classification but on humans.

### Cross-sample and cross-subject training with sample selection

**Figure 4:**
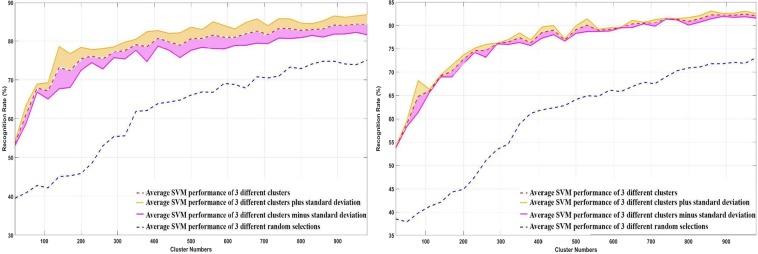
Average of recognition rate of cross-subject (left) and cross-sample (right) training respectively with sample selection in solid red line versus a random selection of data in dashed blue line as a function of the number of images in the training dataset. Yellow and purple lines show the average recognition rate plus and minus standard deviation respectively.

We tested the performance of the handcrafted pipeline when the number of input data is limited. For this approach, images of each state are divided into training and testing sets, and then the training set is split into an increasing number of clusters based on their similarities. We stop at around 1000 clusters when a plateau of performance is reached. Then, a random image of each cluster in each state is selected to train the model, and the model is tested on the test data. Figure 4 shows the average recognition rate of the system after three trials as a function of the number of clusters, i.e., the size of the data set for the training for both cross-subject and cross-sample approaches. As visible in Fig. 4, the performance of both cross-sample and cross-subject training with sample selection overpasses the random selection of images with a gain approximately constant of 13% of recognition rate in all the range. However, at its maximum level, the performance is lower than the best performance obtained in Table 4. This approach can also be used for real-time applications as there is no need to use clustering on test data.

## Methods

### Experimental protocols and associated training strategies

Our main objective is to automate the classification process of mouse tissues into three classes, healthy, inflammation, and cancer tissues. Below, we describe two different medical use cases, where these predictions are helpful. In other words, two different approaches of splitting data into training and testing for our experiments are introduced, which refers to two different clinical problems where prediction is required on subjects or samples.

### Scientific use cases

#### Cross-subject predictions

This use case arises when a prediction must be made on unknown subjects (unknown mice) using a model which has been created (trained) during an off-line training phase. The underlying scientific question addressed by this use case is whether locally acquired samples of tissue can be correctly classified without any additional information from the same subject. Alternatively, in other words, we would like to study whether prediction models based on machine learning can generalize to unseen subjects; it quantifies to which extent the observed diseases are generic or patient-specific.

In a real-world scenario, the corresponding prediction model is static in a sense that different predictions on new subjects will be based on the same model acquired by the medical personnel at a single instant (software updates not with standing). It means a model is trained on a given set of subjects, and will then apply it to new subjects (previously unseen). Decoupling training and prediction is the main advantage of this use case, as the prediction model does not require re-training between predictions, and results can be obtained using the same model on any new subject.

#### Cross-sample predictions

The second use case focuses more on individual tissue samples. This situation arises when one or more subjects are studied in detail, and a large number of tissue samples need to be classified. The underlying scientific question is, whether tissue annotation can be done semi-automatically when a large number of tissues need to be annotated from a low number of subjects. Alternatively, in other words, we would like to study whether a prediction model based on machine learning can generalize to different regions from the same or different subjects.

In a real-world scenario, the corresponding prediction model is dynamic, as (on-line) re-training is necessary for regular intervals. The medical personnel uses an application, which allows them to view tissue samples and annotate them in real-time, available in the additional information section.

The two uses cases are inherently different. Cross-subject predictions are usually more difficult, as the shift between the training data distribution and testing data distribution is generally higher, putting higher requirements on the generalization performance of the predictors. In practice, both cases can be addressed using fully supervised machine learning.

### Proposed training strategies

We propose three different training strategies to address the scientific use cases described above.

#### Cross-subject training

This training strategy is designed to cover the cross-subject use case. The data set is split cross-subject wise, i.e., that subjects (mice) whose samples are in the training set are not present in the test set. It should be considered that the colon’s wall of a subject can sometimes consist of all three labels at the same time, which means that a part of the colon’s wall show cancer tissues. Another part show some inflammation tissues, and the rest can be considered as healthy tissues. Thus, it is essential to design a classifier that tries to label every image independently. Later a subject could be labeled based on the majority of labels of its images.

#### Cross-sample training with all samples

This strategy corresponds to the cross-sample use case. The data set is split into training and test sets by randomly sampling images of each type to be classified (health, inflammation, and cancer). In particular, this approach selects images without information on whether they are consecutive in video frames, or whether they belong to a given subject. In this strategy, images from one subject (a mouse) can be in both training and testing sets, but it does not mean that the same images are used in training and testing. As the microprobe captured images through the colon’s wall of subjects, each image is taken from one specific part (tissue) of the colon’s wall.

#### Cross-sample training with sample selection

In an alternative training strategy for the cross-sample use case, we address the fact that images correspond to video frames which are acquired in the continuity of a local probe inspection process. Therefore, consecutive images are visually similar with a high probability. This temporal correlation between frames can lead to skewed (unbalanced) data distribution and, if not dealt with, to sub-optimal performance.

We propose an unsupervised sample selection processing based on clustering. Features are extracted from each image, which includes standard deviation, mean, variance, and the skewness of the raw pixel values. The features are clustered with k-means, and a single sample is picked from each cluster for training. The rest of the images of the database are used for testing.

### Features, feature learning and classification

Independently of the training strategy, we proposed two different procedures, including both feature extraction and classification methods. The first is based on handcrafted features, whereas the second resort to automatic learning of the intermediate representation.

#### Handcrafted features

In this methodology, we handcraft feature representations instead of learning them. Handcrafted representations have been optimized by the computer vision community over decades of research, including theoretical analysis and experiments. In our setting, we resort to the local binary patterns (LBP)^16^, a state-of-the-art handcrafted descriptor which has been used in a variety of tasks in computer vision, among which are face recognition, emotion recognition, and others, see the survey in^17^. Notably, LBPs have been shown to be valuable for medical image texture analysis^18^.

Under the original form of^16^ and as used in this article, for a pixel positioned at the point $(x,y)$, LBP indicates a sequential set of the binary comparison of its value with the eight neighbors. In other words, the LBP value assigned to each neighbor is either 0 or 1, if its value is smaller or greater than the pixel placed at the center of the mask, respectively. The decimal form of the resulting 8-bit word representing the LBP code can be expressed as follows:

1 $$LBP\,(x,y)=\mathop{\sum }\limits_{n=0}^{7}\,{2}^{n}s({i}_{n}-{i}_{x,y})$$

where ${i}_{x,y}$ corresponds to the grey value of the center pixel, and ${i}_{n}$ denotes that of the ${n}^{th}$ neighboring one. Besides, the function $s(x)$ is defined as follows:

2 $$s(x)=\{\begin{array}{cc}1 & x\ge 0\\ 0 & x < 0\,.\end{array}$$

The LBP operator remains unaffected by any monotonic gray scale transformation, which preserves the pixel intensity order in a local neighborhood. It is worth noticing that all the bits of the LBP code hold the same significance level, where two successive bit values may have different implications. The process of Eq. (1) is realized at the scale of a patch size of $N\times N$ pixels. The $LBP(x,y)$ of each pixel inside this patch are concatenated to create a fingerprint of the local texture around the pixel at the center of the patch. Eqs. (1) and (2) are applied on all patches of an image. Finally, all histogram outputs of patches (after applying LBP on them) are concatenated and considered as the feature vector of an image. This patch size *N*, in this study, is chosen in the order of an average size of vesicular crypts on health images. In our database, a patch size of 8 × 8 can almost cover a healthy vesicular crypt. At the next step, a linear SVM is applied to classify the images based on their LBP features.

### Representation learning

Representation learning, or deep learning, aims at jointly learning feature representations with the required prediction models. We chose the predominant approach in computer vision, namely deep convolutional neural networks^19^, which have proven to be well suited for standard tasks in the medical domain like cell segmentation^20^, tumor detection, and classification^21^, brain tumor segmentation^22^, De-noising of Contrast-Enhanced MRI Sequences^23^ and several other purposes^15^. We train two different models, one which was designed for the task and trained from scratch, and one which has been adapted from (and pre-trained on) image classification.

#### Training from scratch

The baseline approach resorts to a standard supervised training of the prediction model (the neural network) on the target training data corresponding to the respective training strategies described in section. No additional data sources are used. In particular, given a training set comprised of *K* pairs of images ${x}_{i}$ and labels ${\hat{y}}_{i}$, we train the parameters $\theta$ of the network *f* using stochastic gradient descent to minimize empirical risk:

3 $${\theta }^{\ast }={\rm{\arg }}\,\mathop{{\rm{\min }}}\limits_{\theta }\mathop{\sum }\limits_{i=1}^{K}\, {\mathcal L} ({\hat{y}}_{i},\,f({x}_{i},\theta ))$$

${\mathcal L}$ denotes the loss function, which is cross-entropy in our case. The minimization is carried out using the ADAM optimizer^24^ with a learning rate of 0.001.

**Figure 5:**
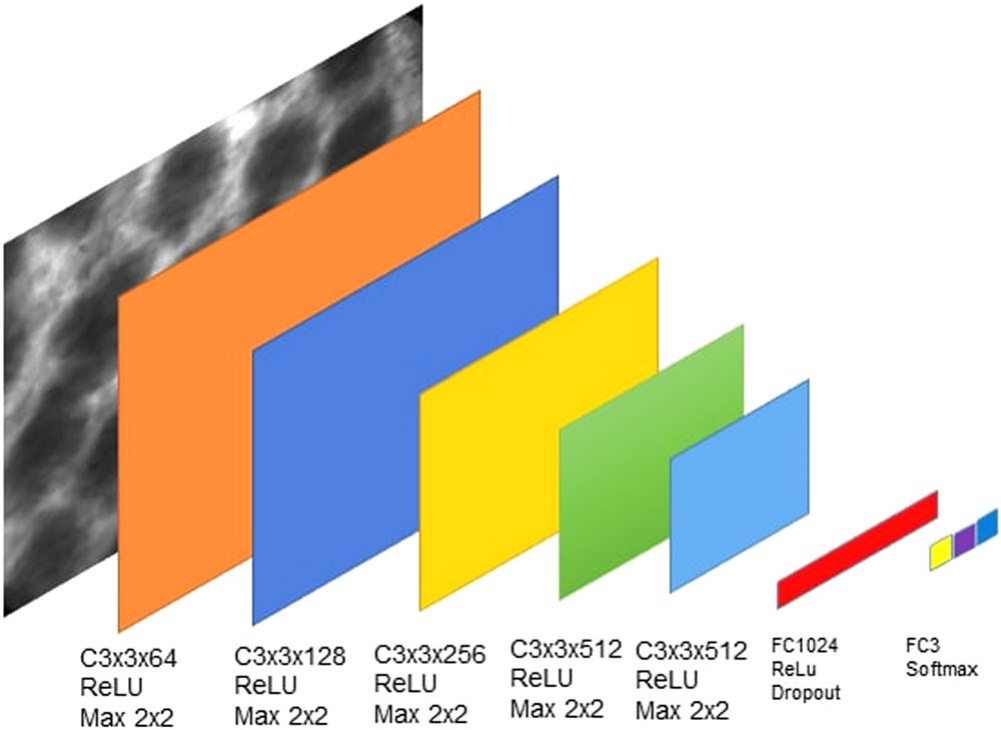
The proposed architecture of the deep network optimized for the task on the cross-validation set.

The architecture of our proposed architecture $f(\cdot ,\cdot )$, shown in Fig. 5, has been optimized on a cross-validation set and is given as follows: five convolutional layers with filters of size 3 × 3 and respective numbers of filters 64, 128, 256, 512, 512 each followed by ReLU activations and 2 × 2 max pooling; a fully connected layer with 1024 units, ReLU activation and dropout (p = 0.5) and a fully connected output layer for 3 classes (health, inflammation and cancer) and softmax activation.

#### Transfer learning

Deep learning addresses complex prediction problems through neural networks with high capacity, i.e., highly non-linear functions with a large number of parameters, whose estimation typically requires a large amount of annotated training data. If this data is not available, the trained networks tend to overfit on the training data and thus generalize poorly to unseen data.

A standard solution to this problem is transfer learning or domain adaptation. The idea is to learn high capacity models on large alternative source data sets whose content is sufficiently correlated with the target application and then transfer the learned knowledge to the target data. Various techniques have been proposed, which differ, among other in the way this transfer is performed and whether labels are available for the target data set (supervised techniques, e.g.^25,26^) or not (unsupervised techniques, e.g.^27^).

We perform supervised transfer using classical weight freezing and fine-tuning^25^, which transfers knowledge by first solving Eq. 3 on the target data set, and then using the obtained parameters ${\theta }^{\ast }$ as initialization (starting point) for the training of the network on the target data set. The assumption is somehow grounded by the existence of standard features in images from natural scenes, which transfer well to images from other domains.

We transfer knowledge from the well-known image classification task ILSCVR 2012 (aka *ImageNet*), a dataset of roughly one million images and 1000 classes^28^. Our model architectures optimized for this task, and as described above, is very likely to underfit on this transfer learning setting. Its hyper-parameters, among which are its architecture and the number of parameters, has been optimized over a validation set, which is very much smaller than the ILVSRC data by roughly a factor of 500. Its design capacity will, therefore, tend to be much too small for the knowledge encoded in the source data (ILVSRC). For this reason, we take “classical” and well-known high-capacity models for the ILVSRC task, namely VGG16^29^, DenseNet^30^, and ResNet50^31^. From the pre-trained model, we remove the task-specific output layer (designed for 1000 classes) and replace it with a new layer for three classes. Among all possible combinations of freezing layers which tested, the model with freezing at the first 3 layers and fine-tuning the other layers on the validation data set returned the best performance shown in the Table 3. The results of the transfer learning method with different freezing layers on our database show the transferability of features from ImageNet database in the spirit of^25^.

We would liketo point out that the two different strategies (training from scratch vs. pre-training and transfer) are compared using two different model architectures. Our goal is to compare strategies, and different strategies can possibly have different optimal architectures. Network architectures need to be adapted to various parameters of the problem, namely the complexity of the task and the number of training samples. As mentioned above, in our case, there is a big difference between the small size of our dataset and the large size of typical computer vision datasets like the ImageNet/ILSVRC dataset (1 M images). Therefore, this involves optimizing parameters (through SGD) as well as the hyper-parameters (through model-search). Only if both are optimized, the potentials of the two strategies are compared. In contrast, comparing two identical architectures would have been inconclusive, as one of two architectures would have been better suited to the task at hand.

### Research involving animals

All applicable international, national, and/or institutional guidelines for the care and use of animals were followed. All procedures performed in studies involving animals were in accordance with the ethical standards of the institution or practice at which the studies were conducted.

### Ethical standards

This study was approved by the institutional review board of the Université Claude Bernard Lyon 1 (reference number: DR2014-62-v1) and complied with ethics committee standards.

### Annotating software

The annotating software tool has been specially developed for this study but is applicable to any video endoscopy annotation for cancer. It is freely available at https://uabox.univ-angers.fr/index.php/s/AZ2IZl6LDYRcd8P together with a demo video and some data sample.

## Database

The experiments involving animals were led in accordance with the rules of the University Lyon 1 Ethics Committee on animal experimentation. Animals were acclimated for two weeks prior to the experiment in the following environment: a 12-hour day/night rhythm in 300 *cm*^2^ plastic cages (for four animals) with straw bedding, pellet food, and tap water. The temperature of each cage was monitored and kept between 19 and 21 C. To induce colitis, mice were chemically treated with a single injection of azoxymethane (AOM, intraperitoneal injection, 10 mg/kg body weight) at the beginning and then, during six months, with dextran sulfate sodium in drinking water (DSS, concentration of 2%). During the experiment, a pressure sensor placed on the mouse’s chest in order to monitor the respiratory index of animals. Analyzed images used in this article chosen at the extrema of the respiratory cycle, where the movements are the slowest to minimize artifacts due to these movements. Mice anesthetized with 3% isoflurane and aspiration flow set at 0.4 L/min during the induction phase. A 25 *μL* solution of Fluorescein Isothiocyanate FITC- Dextran 5% (Sigma Aldrich), used as a contrast agent, is injected in retro-orbital of the mouse’s eye before the CEM investigation.

The anesthesia maintained during imaging with 1.4 to 1.7% isoflurane vaporization and aspiration flow set up on 0.4 L/min. The endoscopic test was conducted using a mini multi-purpose rigid telescope dedicated to small animals (Karl Storz). Acquisition of images made by using a 488 nm confocal endomicroscope CEM (CellVizio c, Mauna Kea Technologies) combined with a 0.95 mm outer diameter Proflex MiniZ microprobe (PF-2173, Mauna Kea Technologies). The microprobe was inserted through the operating sheath of this endoscope and positioned on the mice’s colon walls. During the acquisitions, the depth assessed was approximately 58 *μ*m for a lateral resolution of 3.5 *μ*m and a frame rate of 12 fps. The output image size is 329 × 326 *μ*m^2^ corresponding to a matrix of 292 × 290 pixels^10^.

In total, 38 mice were included in the study for a total of 66788 images which have been annotated as healthy tissue images (6474 images from 9 mice), cancer tissue images (46566 images from 13 mice) or inflammation tissue images (13748 images from 16 mice) by two experts together at the same time with a pre-knowledge of mice diseases. Images were also labeled according to the mice from which they were acquired. Annotation was realized with the help of an application (available in the additional information section) especially developed for this study freely available, as pointed in the supplementary material section. It enables the classification of images according to the three classes studied in this article but also other classes of interestin biomedical studies of the colon’s wall. This application is made available as supplementary material to this study. As mentioned in^5^, some of the raw images do not carry any information for diagnosis. This can be due to misposition of the probe which does not receive enough signal, a decrease of the fluorescence, saturation of the imaging sensor due to too high amount of fluorescence, due to residues, due to contrast agent extravasation or presence of some light-absorbing objects within mucous film located between the probes and the tissue. To prevent the expert from spending time on annotating such non-relevant images and improve the learning process, we decided, as usually done in video endomicroscopy^32,33^ to withdraw them automatically and only keep the informative frame. A simple test based on the computation of the skewness of the gray level histogram of the images demonstrated to be very efficient for this task. Images with a skewness higher than −5 (as an empirical threshold) were kept. The skewness captures the dissymmetry of the histogram around its mean value. This is useful to detect saturated or underexposed images. We estimated, on some 6000 images, that this simple statistical test performs 98% of good detection for the detection of images carrying no useful diagnostic information with a false alarm of 1%. Additionally, in order to assess the influence of theses artifactual images if they would not have been removed, an additional experiment has been done on all raw data (without removing noisy data). This experiment showed a reduction of 2% (on average) on the recognition performance of each training strategy by using our proposed CNN model. This demonstrates the interest of the denoising step but also quantify the robustness of our model.

Based on the training strategies, the database was spilled into three datasets of training (for training of our model), validation (to optimize hyper-parameters), and testing (to report performance on). In the cross-subject training strategy, images of each subject (mouse) were transferred into one of the datasets of training, validation, and testing. The exact number of mice in each dataset shown in Table 1. In the cross-sample training strategy, 75% of the whole database transferred to the training dataset, and the rest of the data belonged to the testing dataset. In this case, the validation dataset was extracted from the training dataset for deep learning experiments. This splitting database approach made a guaranty that the test dataset was not seen during training and validation of the model.

## Conclusion

In this paper, we have presented three classification approaches to classify three states of health, inflammation, and cancer on mice colon’s wall. Fully automated machine learning-based methods are proposed, including deep learning, transfer learning, and classical texture-based classification. Different training strategies are compared in order to find the best approach for this specific problem. The images processed in this paper were acquired in the framework of a preclinical study on colon mice. In this type of study (preclinical), the size of the database is not comparable with other domains in machine learningAs also underlined in^34^ on the different types of images, we found that a custom deep learning model shows superiority over handcrafted features and well-known deep learning-based architectures. The best classification performance on this type of images are achieved with our proposed CNN model which are trained on colon’s wall images.

In the cross-sample case, where generalization to unseen subjects is not an issue, Deep learning gave a performance of 99.93% of correct classification. Similar to the cross-sample, in the cross-subject approach where classification on un-seen objects is an issue, our proposed CNN method showed a performance of 98.49% of correct classification. These are usual order of magnitude of performance obtained with nowadays machine learning approaches when vast data sets are available, but this can be considered as excellent performance indeed here since we worked with the typical small data sets available in preclinical studies.

This work corresponds to the first fully automated classification algorithm for mice colon’s wall images reported in the literature. Similar works were carried on the human colon’s wall with the same imaging system. The comparison of the closest work^14^ with our algorithm shows a comfortable margin of a 14% of accuracy. This is an interesting result which demonstrates that in the perspective of machine learning, there is no guarantee of translational research between human and animal. Also, a novel unsupervised sampling strategy based on the specific similarities of images in the acquisition of images with endomicroscopy in the colon has been designed. The interest of this sampling strategy has been demonstrated in terms of amount of data required in the training data sets to reach a plateau of performance. However, the performance of this sampling strategy is lower than brute forces classical approaches. It would be possible to improve the metric of similarity used to select the images in the training data sets automatically. This was based on first-order statistics in this study, but other approaches could be used to include more dynamical information. However, due to the multi-scale sources of temporal noise (movement of the probes^35^, passing of unexpected items between probe and tissues, biological movement,etc.) it would be an open question to determine a reasonable time scale for this smoothing.

Our clustering method is somewhat related to active learning, where the agent requests feedback on data from a user. The comparison is a little bit a stretch, as no new data is collected from decisions by an agent. In our current implementation, the dataset stays stable, and only a subset is actively chosen.

However, we plan to investigate active learning as future work, where a classifier is trained on a subject followed by continued examination of the subject on new samples. Here, an agent could quickly provide decisions on (i) which samples should be added to the training set, and (ii) into which direction the user should emphasize its search in order to optimize performance and discovery. This leads to an exploitation/exploration trade-off known from Reinforcement learning.

Direct perspectives of other sampling strategies are possible. It would now be possible to apply the classification scheme developed here to produce a score on individual mice quantifying the number of images with the disease. Such a quantification could then be compared with clinical scores realized on other types of imaging systems in a multimodal perspective such as the one recently shown with magnetic resonance imaging^36^. Also, the machine learning approach presented with a discussion on the different training strategies could be transposed to other bioimaging problems. In confocal endomicroscopy, this includes, for instance, the characterization of other colon’s diseases observed in confocal microscopy^37^ or other parts of the digestive system^38^ or also to other organs^39^ which have received interest and could benefit from machine learning approaches to perform automated characterization of tissues.
